# Advances and barriers in gender equity within Brazilian rheumatology: a two-year follow-up study

**DOI:** 10.1186/s42358-026-00566-8

**Published:** 2026-07-17

**Authors:** Mariana Peixoto Guimarães Ubirajara e Silva de Souza, Ricardo Machado Xavier, Odirlei Monticielo, Latika Gupta, Pavel Ovseiko, Maria Fernanda Guimarães, Grace Wright, Laura Coates, Vikas Agarwal, Viviane Angelina de Souza, Adriana Maria Kakehasi

**Affiliations:** 1 Post Graduate Program in Sciences Applied to Adult Health Care, Federal University of Minas Gerais, Belo Horizonte, Minas Gerais Brazil; 2 Federal University of Rio Grande do Sul, Porto Alegre, Brazil; 3 Federal University of Rio Grande do Sul, Porto Alegre, Brazil; 4 Department of Rheumatology, Royal Wolverhampton Hospitals NHS Trust, Wolverhampton, UK; 5 Department of Inflammation and Ageing, School of Infection, Inflammation and Immunology, University of Birmingham, Francis Crick Institute, London, UK; 6 Radcliffe Department of Medicine, University of Oxford, Oxford, UK; 7 Federal University of Minas Gerais, Belo Horizonte, Brazil; 8 Association of Women in Rheumatology, 345 E 37th Street, Suite 303C, New York, NY 10016 USA; 9 Nuffield Department of Orthopaedics, Rheumatology and Musculoskeletal Sciences, University of Oxford, Oxford, UK; 10 Department of Clinical Immunology and Rheumatology, Sanjay Gandhi Postgraduate Institute of Medical Sciences, Lucknow, India; 11 Rheumatology, Federal University of Juiz de Fora, Juiz de Fora, Minas Gerais Brazil

**Keywords:** Gender equity, Rheumatology, Brazil, Survey, Career advancement, Work-family interface, Harassment

## Abstract

**Introduction:**

Gender equity is increasingly recognized as essential to integrity and sustainability of the medical workforce, yet significant disparities remain. This study aims to evaluate longitudinal trends in gender equity within Brazilian rheumatology, focusing on career advancement, work-family interface, and workplace safety.

**Methods:**

This comparative cross-sectional study analyzed two self-administered, web-based surveys conducted at different moments among members of the Brazilian Society of Rheumatology (SBR), to assess temporal changes. The surveys included Reuma Equity (2022, *n* = 459, 67.1% women) and the Brazilian subset of the global Coalition for Health and Gender Equity (CHANGE) (2024, *n* = 301, 74.4% women). Statistical analyses included Pearson’s chi-square, Fisher’s exact, and Mann–Whitney tests, with a significance level set at *p* ≤ 0.05.

**Results:**

In both moments, female rheumatologists were younger [2022: OR 3.7, (95% CI 2.2–6.4, *p* = 0.001); 2024: OR 4.3, (95% CI 2.2–8.6, *p* < 0.001)], carried a greater burden of domestic labor [2022: OR 2.8, (95% CI 1.8–4.3, *p* < 0.001); 2024: OR 2.8, (95% CI 1.5–5.1, *p* < 0.001)], and men were more likely to occupy prominent positions at national congresses [2022: OR 2.3, (95% CI 1.5–3.4, *p* < 0.001); 2024: OR 2.1, (95%CI 1.3–3.6, *p* = 0.005)]. There was an increase in the probability of women being more likely to refuse career progression for family-related reasons [2022: OR 2.1, (95% CI 1.2–3.7, *p* = 0.038); 2024: OR 3.2, (95% CI 1.8–6.1, *p* < 0.001)], and a decrease in reported rates of sexual harassment [2022: OR 4.2, (95% CI 2.5–6.9, *p* < 0.001); 2024: OR 2.6, (95% CI 1.4–4.6, *p* = 0.002)]. Gender disparities in medical visit fees (lower fees: 69.2% women and 57.6% men, *p* = 0.043), and medical visit duration (longer appointments: 78.2% women and 69.5% men, *p* = 0.014) observed in 2022 disappeared by 2024 (*p* = 1.00, *p* = 0.318).

**Conclusion:**

Although representation in Brazilian rheumatology is increasing, women continue to shoulder disproportionate domestic responsibilities, decline career opportunities more often, and experience higher rates of harassment. Even so, improvements were observed in professional indicators and in the male’ views, suggesting gradual progress toward gender equity.

**Supplementary Information:**

The online version contains supplementary material available at https://doi.org/10.1186/s42358-026-00566-8.

## Introduction and Background

 The pursuit of gender equity has become a central focus for modern society, recognized as a vital component of both research integrity and medical workforce sustainability [1]. While the term gender equality refers to the provision of identical opportunities for all individuals to participate in public life, gender equity involves the fair treatment of individuals according to their specific needs to achieve equivalent career outcomes [2].

Despite significant increases in university attendance and workplace participation among women, global labor statistics indicate that substantial disparities persist, particularly in leadership and high-level managerial roles [3, 4]. This phenomenon is especially visible in academic medicine, where female physicians often face systemic barriers such as reduced remuneration, limited access to leadership grooming, and lower publication-related productivity metrics compared to their male counterparts [5].

In Rheumatology, although there is a notable trend toward a female-predominant workforce among trainees and early-career practitioners, women continue to be a minority in senior academic positions, international congress chairing roles, and editorial board memberships [6–8].

These disparities are often attributed to the “motherhood penalty,” where the unequal distribution of domestic labor and caregiving responsibilities restricts a woman’s ability to pursue research funding and career progression. Furthermore, workplace safety remains a significant hurdle; global data suggests that female rheumatology professionals are nearly four times more likely to experience sexual harassment and twice as likely to face bullying compared to their male colleagues [8, 9].

While specialties such as surgery and cardiology have established frameworks for monitoring gender disparities, rheumatology has historically lacked large-scale, multinational assessments of the lived experiences of its professionals. To address this evidence gap, the Reuma Equity Project assessed the Brazilian reality regarding gender equity in rheumatology in 2022 [9]. Since this project, Brazilian Society of Rheumatology (SBR), included the gender equity subject in the national congress and local forum presenting preliminaries data and promoting discussions. Two years later, Brazil also participated of the Coalition for Health and Gender Equity (CHANGE) to characterize the global landscape of equity through comprehensive survey data [8]. This study aims to evaluate longitudinal trends in gender equity within Brazilian rheumatology, focusing on career advancement, the work-family interface, and workplace safety.

## Methods

The Reuma Equity Project was a cross-sectional study conducted using a self-administered, web-based survey in 2022, among members of the Brazilian Society of Rheumatology (SBR), disseminated through mailing lists and social media channels. The survey consisted of 33 questions covering academic sociodemographic, work performance, work-family interface, gender inequity or harassment, and improvement possibilities.

CHANGE study was a cross-sectional, self-administered web-based survey of rheumatologists and allied health professionals, disseminated through professional organizations, mailing lists and social media channels, conducted between January 2023 and May 2024, translated into eight languages (English, Spanish, Japanese, Portuguese, Thai, Traditional Chinese, Simplified Chinese, and French) in 105 countries. In Brazil, CHANGE questionnaire was distributed in August and September 2024, only to SBR’s rheumatologists. The 38-item survey covered academic sociodemographic, work performance, gender discrimination, bullying, sexual harassment, work-family interface, career progression, and interventions [10].

Both questionnaires were voluntary, participants were not identified, and no reward was offered. Incomplete responses to interventions or demographics data, were excluded from the analysis.

The medical visits fees in the questionnaires were converted from Brazilian reais to US dollars, using the exchange rate of 1 USD = R$ 5.48 in effect during the period. The current study analyzed the data from the two surveys, with the aim of assessing changes over time. The results are reported according to the “Strengthening The Reporting of Observational Studies in Epidemiology” (STROBE) guidelines [11]. The comparisons between the two time points are descriptive. The questionnaires used in the Reuma Equity and Change studies were included in the supplementary material (Appendix 2 and 3).

### Statistical analysis

The analyses were performed using R version 4.4.0, PASW Statistics 18, and MINITAB. A significance level of 5% was used. Descriptive statistics were used to summarize demographic characteristics and intervention preferences. The results were obtained using measures of central tendency (mean and median) and measures of dispersion (standard deviation) for quantitative characteristics, and by means of frequency and percentage for qualitative characteristics. Comparisons between the qualitative characteristics and sex were performed using Pearson’s Chi-square test or Fisher’s exact test (in cases with at least one expected frequency less than 5). Associations were located and quantified from comparisons between pairs of categories, correcting the significance level using the Bonferroni method. The associations were quantified by the Odds Ratio (OR) and their respective 95% confidence intervals (IC). Comparisons between the quantitative characteristics and sex were performed using the Mann-Whitney test, since the usual assumptions (normality and/or homoscedasticity) were violated. Normality was assessed using the Kolmogorov-Smirnov test and homoscedasticity using the Levene test. Associations were quantified by differences in medians and their respective IC 95%.

## Results

### Sociodemographic profile

The 2022 Reuma Equity survey included 459 participants, of whom 308 (67.1%) were women and 151 (32.9%) were men, representing 21.7% of SBR members. The majority were under 51 years, being 91 (19.7%) between 22 and 36 years, 202 (43.8%) between 37 and 51 years, 96 (20.8%) between 52 and 62 year and 72 (15.6%) over 62 years; with women being more likely to be in the younger age group [OR 3.7 (95% CI 2.2–6.4, *p* = 0.001)].

CHANGE survey in 2024 included 1,945 participants in 105 countries, with 304 (15.6%) Brazilian respondents. Three participants who answered as their gender, “prefer not to say”, “transgender women”, “non-binary person”, were excluded from Brazilian statistical analysis due to low representativeness, totalizing 301 valid answers. Of these, 224 (74.4%) were women and 77 (25.6%) men. The majority were also under 51 years, being 71 (23.6%) between 22 and 36 years, 134 (44.5%) between 37 and 51 years, 54 (17.9%) between 52 and 62 year, 38 (12.6%) over 62 years, and 4 (1.3%) didn’t answer; with women being more likely to be in the younger age group [OR 4.3 (95% CI 2.2–8.6, *p* < 0.001)]. Over 50% of participants in both surveys lived in Southeast region of Brazil (Table 1).

**Table 1:** Sociodemographic data from Reuma Equity and change, by gender

	Reuma Equity			Change		
	Women (%)	Men (%)	*p*-value	Women (%)	Men (%)	*p*-value
**Gender**	308 (67.1)	151 (32.9)		224 (74.4)	77 (35.6)	
**Years**						
Under 51	215 (69.8)	76 (50.3)	**< 0.001**	162 (73.0)	43 (57.3)	**< 0.001**
Between 52–61	62 (20.1)	34 (22.5)		40 (18.0)	9 (12.0)	
Over 62	31 (10.1)	41 (27.2)		20 (9.0)	23 (30.7)	
**Region of residence**						
North	10 (6.2)	7 (4.6)		10 (4.5)	0 (0)	
Northeast	45 (14.6)	25 (16.6)		35 (15.6)	14 (18.2)	
Midwest	40 (13)	12 (8)		23 (10.3)	8 (10.4)	
Southeast	162 (52.6)	81 (53.6)		123 (54.9)	44 (57.1)	
South	42 (13.6)	26 (17.2)		31 (13.8)	10 (13)	
Not Reported	0 (0)	0 (0)		2 (0.9)	1 (1.3)	

### The work-family interface

Across both cohorts, the demographic profile remained consistent: female rheumatologists were twice as likely to be the legal guardians of minors under 18, [2022: OR 2.2 (95%CI 1.5–3.3, *p* < 0.001)]; [2024: OR 1.9 (95% CI 1.1–3.2, *p* = 0.025)], with parental leave of 4 months or over presented predominantly by women (*p* < 0.001). In both 2022 and 2024, women were significantly more likely to report being solely or predominantly responsible for household chores [2022: OR 2.8 (95% CI 1.8–4.3, *p* < 0.001)]; [2024: OR 2.8 (95% CI 1.5–5.1, *p* < 0.001)] when compared to equitable distribution or the household chores being made predominately or solely by men.

In both time points, but increased over time, women significantly more often reported refusing career progression for family reasons [2022: OR 2.1 (95% CI 1.2–3.7, *p* = 0.038); [2024: OR 3.2 (95% CI 1.8–6.1, *p* < 0.001)], as well as the perception that family demands changed the course of one’s career [2022: OR 2.1 (95% CI 1.2–3.5, *p* = 0.005)]; [2024: OR 4.5 (95% CI 2.1–9.6, *p* < 0.001)]. This question changed format between the two moments. The interference of work duties with family planning was statistically significant in [2022 OR 1.8 (95% IC 1.2–2.8, *p* = 0.002)] but lost significance in the 2024 cohort (*p* = 0.168) (Tables 2 and 3).

**Table 2:** Comparisons of domestic responsibilities load, by gender (concordant responses shown)

	Reuma Equity			Change		
	Women (%)	Men (%)	*p*-value	Women (%)	Men (%)	*p*-value
**Legal guardian of someone under 18?**	162 (52.7)	51 (33.8)	**< 0.001**	110(49.5)	26 (34.7)	**0.025**
**Maternity/paternity leave?**						
More than 4 months	100 (44.5)	0 (0.0)	**< 0.001**	74 (52.5)	0 (0.0)	**< 0.001**
Less than 4 months	128 (56.1)	46(100)		67 (47.5)	29 (100)	
**Household chores performed by**:						
Women	185 (64.0)	59 (41.3)	**< 0.001**	137(64.0)	28 (38.4)	**< 0.001**
Equally (men/ women)	77 (26.6)	68 (47.5)		72 (33.7)	41 (56.1)	
Men	27 (9.4)	16 (11.2)		5 (2.3)	4 (5.5)	

**Table 3:** Comparisons of the relationship between career-family interface, by gender (concordant responses shown)

	Reuma Equity			Change		
	Women (%)	Men (%)	*p*-value	Women (%)	Men (%)	*p*-value
**Decline career advancements for family reasons (agree)**	164 (53.3)	65 (43.1)	**0.038**	131(63.9)	26 (35.6)	**< 0.001**
**Changes in family planning due to work (agree)**	185 (60.1)	65 (43.0)	**0.002**	143(67.8)	37 (55.2)	0.168
**Family demands (including kids) changed the career**						
Agree	247 (80.2)	100 (66.2)	**0.005**			
Positive impact				27 (17.9)	24 (45.3)	**< 0.001**
Negative impact				23 (15.2)	3 (5.6)	
**Job demands interfere family life (agree)**	225 (73.0)	102 (67.6)	0.391	135 (60.3)	40 (51.9)	0.418
**Job time difficult family responsibilities (agree).**	192 (62.3)	76 (50.3)	**0.039**	142 (63.4)	35 (45.4)	**0.010**
**Family’s demands negatively impact work (agree).**				61 (27.2)	10 (13.0)	**0.035**

### Professional development and leadership

**Fig. 1:**
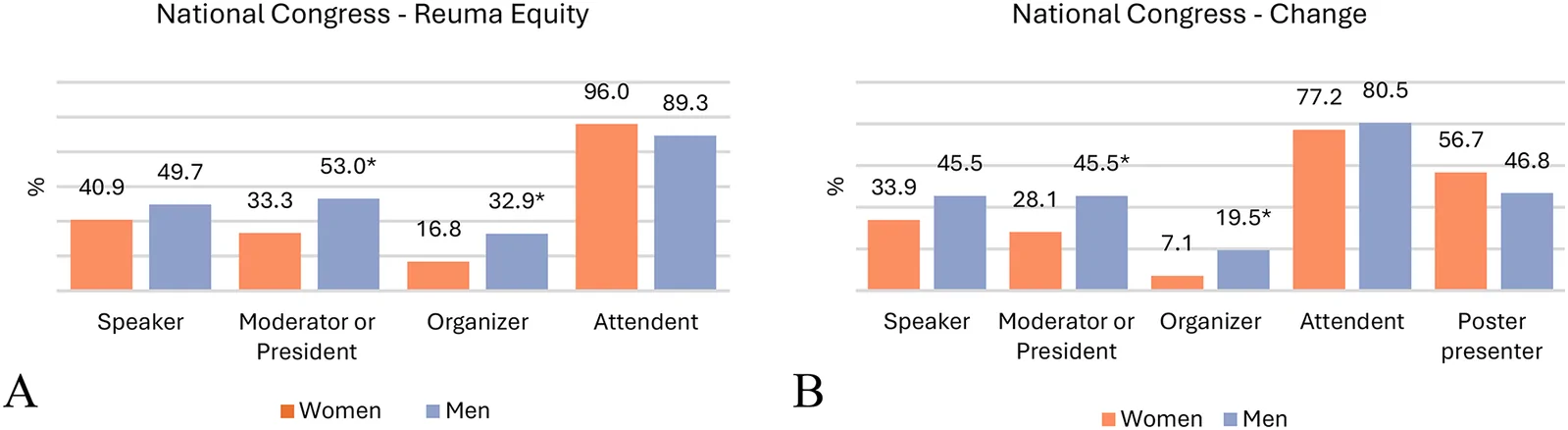
Representativeness in National Congress, by gender. **A**: Reuma Equity. **B**: Change. The presence of the asterisk (*) shows statistical significance with *p* < 0.005

Men were consistently more likely to occupy high-visibility roles in national rheumatology events, such as moderators or presidents [2022: OR 2.3 (95% CI 1.5–3.4, *p* < 0.001)]; [2024: OR 2.1 (95%CI 1.3–3.6, *p* = 0.005)], or organizers [2022: OR 2.4 (95% CI 1.5–3.8, *p* < 0.001)]; [2024: OR 3.2 (95% CI 1.5–6.7, *p* = 0.002)], but not as speakers in both moments (*p* = 0.078; *p* = 0.071). In both moments, men reported significantly more years of professional experience, a finding that aligns with their older age distribution, although academic graduation level, academic function, and leadership position had no statistical difference among genders. In 2022, there were significant gender differences in medical visits fees and duration. Among women, 69.2% received lower fees compared to 57.6% of men (*p* = 0.043), and 78.2% of women had longer medical visits compared to 69.5% of men (*p* = 0.014); however, by 2024, these differences were no longer statistically significant (*p* = 1.000; *p* = 0.318). Participants who worked exclusively in the public sector were excluded from the financial analysis. Following the same tendency, in 2022, women had 2 times more chances to work in public service [OR 2.0 (95% CI 1.3–3.1, *p* = 0.002)] and worked fewer hours during the week (*p* = 0.008), data that was not found in 2024. (Table 4; Figs. 1A/B, 2 A/B).

**Table 4:** Comparison of characteristics related to professional practice, by gender

	Reuma Equity			Change		
	Women (%)	Men (%)	*p*-value	Women (%)	Men (%)	*p*-value
**Medical visit fee**						
< USD 73	213 (69.2)	87 (57.6)	**0.043**	120 (82.7)	49 (83.0)	1.000
USD 73–146	77 (25.0)	54 (35.8)		23 (15.9)	10 (17.0)	
> USD 146	18 (5.8)	10 (6.6)		2 (1.4)	0 (0.0)	
**Medical visit duration**						
< 15 min	0 (0.0)	5 (3.3)	**0.0142**	1 (0.4)	2 (2.6)	0.318
15 to 30 min	67 (21.8)	41 (27.2)		138 (61.6)	48 (62.3)	
30 to 45 min	126 (40.9)	60 (39.7)		55 (24.6)	20 (26)	
45 to 60 min	87 (28.2)	35 (23.2)		30 (13.4)	7 (9.1)	
> 60 min	28 (9.1)	10 (6.6)				
**Work sector (public)**	119 (38.6)	36 (23.8)	**0.002**	79 (35.3)	18 (23.4)	0.118
**Workload weakly**						
< 20 h	14 (4.5)	10 (6.6)	**0.0081**	6 (2.7)	3 (3.9)	0.925
20–44 h	162 (52.6)	66 (43.7)		115 (51.3)	38 (49.3)	
45–60 h	105 (34.1)	46 (30.5)		77 (34.4)	27 (35.1)	
> 60 h	27 (8.8)	29 (19.2)		26 (11.6)	9 (11.7)	

**Fig. 2:**
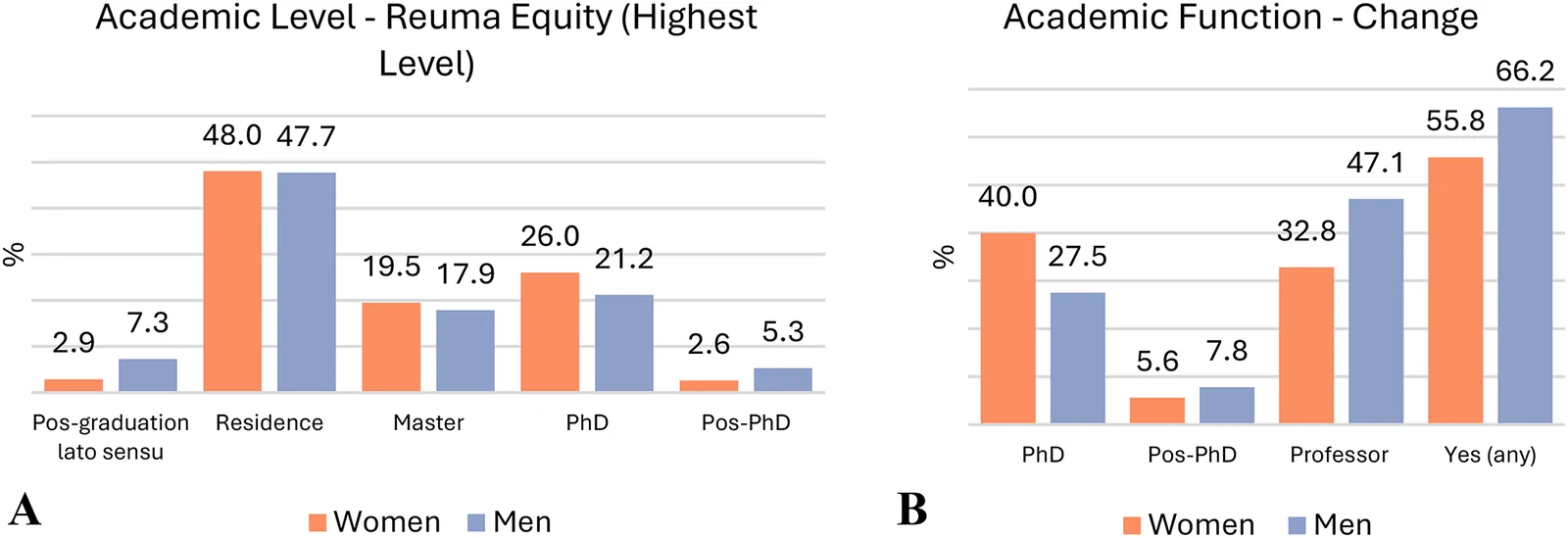
Academic development and function, by gender. **A**: Reuma Equity. **B**: Change

Regarding professional self-confidence, the 2022 survey showed that women reported higher levels of agreement in 5 of 8 measures of resourcefulness and preparedness (*p* < 0.05). In contrast, in the 2024 survey, a significant gender difference was observed in only 1 of the 8 measures, favoring men (*p* = 0.004) (Table 5).

**Table 5:** Comparison of professional self-confidence, by gender (concordant responses shown)

	Reuma Equity			Change		
	Women (%)	Men (%)	*p*-value	Women (%)	Men (%)	*p*-value
Thanks to my resourcefulness. I know how to handle unexpected situations at work	292 (94.8)	138 (91.4)	**0.035**	136 (60.7)	45 (58.4)	0.725
If I have problems at work. I can usually think of something to do:	301 (97.7)	145 (96.0)	0.578	142 (63.4)	48 (62.3)	0.869
I remain calm when facing difficulties at work because I trust my abilities.	285 (92.5)	140 (92.7)	0.647	135 (60.3)	54 (70.1)	0.122
When I encounter a problem at work. I usually find several solutions:	275 (89.3)	132 (87.4)	0.109	109 (48.7)	52 (67.5)	**0.004**
No matter what comes up at my job. I’m usually able to handle it.	272 (88.3)	123 (81.5)	**0.014**	80 (35.7)	29 (37.7)	0.759
My past work experiences have prepared me well for my future career:	293 (95.1)	140 (92.7)	**0.030**	124 (55.4)	43 (55.8)	0.941
I achieve the goals I set for myself in my work:	283 (91.9)	128 (84.8)	**0.005**	125 (55.8)	45 (58.4)	0.687
I feel prepared to meet most of the demands in my job:	297 (96.4)	137 (90.7)	**0.034**	147 (65.6)	56 (72.7)	0.251

### Workplace adversity and discrimination

Reported verbal sexual harassment remained significantly higher among women in both studies [2022: OR 4.2 (95% CI 2.5–6.9, *p* < 0.001)]; [2024: OR 2.6 (95% CI 1.4–4.6, *p* = 0.002)] but showed a reduction in tendency over time. In 2022, women also reported higher rates of non-verbal [OR 5.1 (95% CI 3.2–8.2, *p* < 0.001)] and physical harassment [OR 2.5 (95% CI 1.4–4.7, *p* = 0.003)], that were not included in CHANGE survey.

Specifically evaluated in the 2024, women reported significantly higher rates of bullying [OR 2.3 (95% CI 1.3–4.1, *p* = 0.003)], with no men reporting “frequent or constant” victimization. Overall scores for gender discrimination in professional practice were significantly higher for women in both periods. In 2022, women had markedly greater odds of reporting discrimination [OR 13.8 (95% CI 1.9–102.6, *p* < 0.001)]. In 2024, discrimination scores remained higher among women (mean 1.8 ± 0.7) compared with men (mean 1.1 ± 0.3; *p* < 0.001), with 52.5% of women reporting some frequency of discrimination by older colleagues.

As detailed in the Reuma Equity survey, multiple forms of gender discrimination were identified. Women reported significantly higher rates of discrimination in the academic environment (*p* < 0.001), during job interviews [OR 15.5 (95% CI 2.1–115.2, *p* < 0.001)], receiving lower pay for the same work [OR 5.7 (95% CI 1.7–19.1, *p* = 0.001)], being overlooked for promotion opportunities [OR 3.3 (95% CI 1.0–11.2, *p* = 0.001)], and not obtaining leadership positions [OR 4.2 (95% CI 1.2–14.1, *p* = 0.013)]. Additionally, three participants who identified their gender as “prefer not to say,” “transgender woman,” or “non-binary person” also reported experiences of being assigned tasks below their level of expertise, as well as episodes of gender-based discrimination, sexual harassment, and bullying, occurring with frequencies ranging from “rarely” to “often.” (Table 6).

**Table 6:** Comparison of sexual harassment, bullying and gender discrimination, by gender

	Reuma Equity			Change		
	Women (%)	Men (%)	*p*-value	Women (%)	Men (%)	*p*-value
**Sexual Harassment**						
Verbal	137 (44.5)	25 (16.6)	**< 0.001**	99 (44.2)	18 (23.7)	**0.002**
Nonverbal	166 (53.9)	29 (19.2)	**< 0.001**			
Physical	74 (24)	17 (11.3)	**0.003**			
Work environment						
**Bullying (positive answers)**				83 (37.1)	13 (16.9)	**0.001**
**Gender discrimination**						
Co-workers (mean ± SD)				1.8 ± 0.7	1.1 ± 0.3	**< 0.001**
Work environment (mean ± SD)				1.6 ± 0.7	1.1 ± 0.3	**< 0.001**
Job interview	29 (9.4)	1 (0.7)	**< 0.001**			
Academic environment	54 (17.6)	3 (2.0)	**< 0.001**			

### Perception of gender equity interventions

Regarding interventions perceived as beneficial for promoting gender equity, all 17 items in the Reuma Equity survey showed significantly higher support from women. In contrast, in CHANGE survey, only 9 of the 29 items demonstrated gender differences, while the remaining 20 showed no significant difference between men’s and women’s responses.

**Fig. 3:**
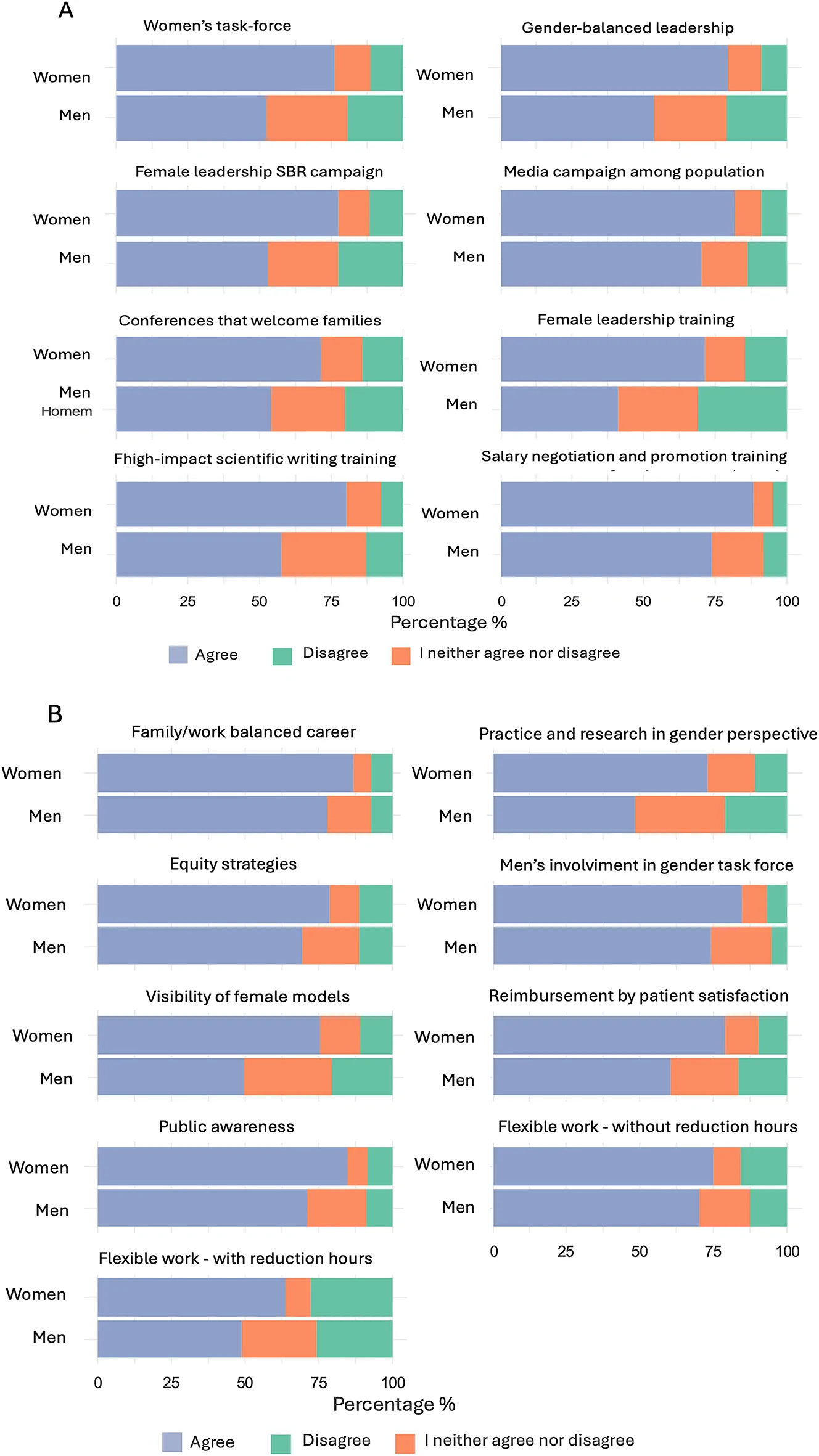
**A/B**: Proposals for interventions promoting gender equity, answers by gender, in Reuma Equity

The items with significant gender differences in the CHANGE survey included: support for gender-balanced leadership within the SBR and its official journal, *Advances in Rheumatology* (AIR) (*p* = 0.031); support for SBR media campaigns highlighting female leadership (*p* = 0.004); assistance in preparing research grant proposals (*p* = 0.042); career-planning training that integrates family - work balance (*p* = 0.016); involvement of men in the gender equity task force (*p* = 0.011); increased visibility of female role models (*p* < 0.001); enhanced public awareness of gender equity (*p* = 0.026); and flexible working hours (or reduced work hours) to accommodate family needs (*p* = 0.026).(Fig. 3A/B). The complete list of proposals suggested in favor of gender equity and the result of agreement in each survey is shown in supplementary material (Appendix 4).

## Discussion

The landscape of gender equity in Brazilian rheumatology was evaluated through two major cross-sectional surveys: the Reuma Equity study in 2022 (*n* = 459) and the Brazilian subset of the global CHANGE survey in 2024 (*n* = 301). A comparative analysis of these datasets reveals persistent systemic disparities alongside some evolving trends in professional practice [8, 9]. While the Brazilian medical landscape is undergoing a rapid feminization—with women becoming the majority of physicians in 2025 (50.9%) - our data confirms that a demographic shift alone does not guarantee gender equity in career advancement or workplace safety [12, 13].

A persistent theme across both cohorts is the disproportionate impact of domestic responsibilities on female professionals. Women were significantly more likely to be the primary guardians of minors and solely responsible for household chores in both years. This domestic inequity translates directly into professional barriers: women were over three times more likely than men to refuse career progression for family reasons with a significant increase from 2022 (OR 2.1) to 2024 (OR 3.2). This phenomenon aligns with the “motherhood penalty” described in global literature, where caregiving duties restrict a woman’s ability to pursue research funding and leadership milestones, while men often benefit from a “fatherhood bonus” in professional settings [14].

In 2022, women showed a reduced weekly workload and higher frequency in public service work. These associations could be attributed to greater job security in the public sector, the possibility of paid maternity leave, and the fact that women have a lighter professional workload, given their greater domestic workload. However, these statistical differences were not evident in 2024, suggesting a possible shift in women’s attitudes towards equity.

Even though Brazilian female rheumatologists reported similar leadership positions and similar academic backgrounds, showing that women persist in the pursuit of individual professional growth, despite an environment of inequality. Men were consistently more likely to occupy positions of moderator, president, or organizer in national events, but not as speakers, highlighting a barrier that is possibly social, with no technical implications. In accordance with international studies, women are underrepresented in the high-impact journal’s citations and editorial boards [5, 7, 15]. The bibliometric analysis of AIR was conducted by our group, showed that women hold most first-author positions (59%) but are significantly less frequent as senior authors (44.4%) and are absent from Editor-in-Chief roles (unpublished data). These results suggest that while women are highly productive at the start of their careers, systemic barriers prevent them from reaching the highest career position.

The data regarding workplace safety remains concerning. The study showed female rheumatologists reported significantly higher rates of sexual harassment, bullying and workplace hostility. These findings suggest that harassment is not an isolated occurrence but a systemic issue that affects women, regardless of their training level. Despite persistently high levels, the study showed a reduction in the rate of harassment, which may be a consequence of the increased visibility, discussion, and involvement of the SBR in the issue of gender equity.

One of the most encouraging findings from the comparison is the normalization of clinical practice metrics. In 2022, significant gender-based differences were observed, with women charging lower medical visit fees, spending more time per patient, having a reduced weekly workload. By 2024, these differences were no longer statistically significant. This shift may indicate a growing alignment in professional valuation and practice standards across genders, possibly driven by increased awareness of equitable remuneration [16].

Finaly, the comparison also reveals a shift in male thinking regarding strategies to mitigate gender inequities. In Reuma Equity survey all proposed changes received significant positive responses for women; however, in 2024, most proposals also received agreement from men, showing no statistically significant difference between genders. This suggests a possible increase in awareness and involvement among men in the pursuit of greater gender equity in Brazilian rheumatology.

To our knowledge, this is the first study to compare gender equity in global rheumatology at two distinct points in time. The changes found may reflect greater visibility and discussion of the topic within Brazilian rheumatology. The large number of participants in Reuma Equity and the high representation of Brazil in the CHANGE cohort, comprising 15.6% of the global sample, increases the robustness of the analysis. The main limitation of the study is the voluntary participation in a self-administered questionnaire. This can introduce selection bias, since people with greater involvement in the subject agree to participate and the responses reflect participant’s personal perception. The cross-sectional design and the differences between the questionnaires limited statistical analysis between the two time points and causal inference.

## Conclusion

The comparative analysis of the Reuma Equity (2022) and CHANGE (2024) surveys provides a longitudinal perspective on the evolving landscape of gender equity in Brazilian rheumatology. The comparison highlights that while demographic representation is increasing, structural barriers persist. Women are still significantly more likely to bear the primary burden of domestic labor, are more likely to refuse career progression for family reasons, hold fewer leadership positions in conferences and academic journals and suffer more harassment. Conversely, differences in medical visits characteristics and a shift in male thinking regarding strategies to mitigate gender inequities, suggest a possible improvement in society.

## Supplementary Information

Below is the link to the electronic supplementary material.

Supplementary Material 1

Supplementary Material 2

Supplementary Material 3

Supplementary Material 4

## Data Availability

All data generated or analysed during this study are included in this published article and its supplementary information files. The dataset used and/or analysed during the current study is available from the corresponding author on reasonable request.

## Abbreviations

AIR: Advances in Rheumatology
CHANGE: Coalition for Health and Gender Equity
CI: Confidence interval
OR: Odds Ratio
SBR: Brazilian Society of Rheumatology
