# Supplementary material for: Advances and barriers in gender equity within Brazilian rheumatology: a two-year follow-up study
Source: Adv Rheumatol. 2026 Jul 17;66:58. doi: 10.1186/s42358-026-00566-8 (PMC13663116; doi:10.1186/s42358-026-00566-8)
Supplement: Supplementary file 1 [file 42358_2026_566_MOESM1_ESM.pdf]

**Appendix 1 - Coalition for Health and Gender Equity (CHANGE) Study Group – Author List**


1. Latika Gupta: 1. Department of Rheumatology, Royal Wolverhampton Hospitals NHS Trust, Wolverhampton, UK. 2. Division of Musculoskeletal and Dermatological Sciences, Faculty of Biology, Medicine and Health, Manchester Academic Health Science Centre, The University of Manchester, Manchester, United Kingdom. ORCID: 0000-0003-2753-2990.
2. Alejandra Magdalena Babini- Rheumatology Unit, Hospital Italiano de Cordoba, Cordoba, Argentina.
3. Ana Maria Arredondo Gonzalez Colombian Association of Rheumatology, Bogotá, Colombia; 2. Department of Rheumatology, Faculty of Medicine, Fundación Universitaria de Ciencias de la Salud (FUCS), Bogotá, Colombia
4. Anne-Marie Russell, Rheumatology, Aarhus University Hospital, Aarhus, Denmark annetrol@rm.dk.
5. Asgar Ali Kalla- Chief of Rheumatology Unit, Groote Schuur Hospital.kalla@iafrica.com
6. Carlos Enrique Toro Gutiérrez, General Director, Reference Center for Osteoporosis, Rheumatology and Dermatology, Pontifica Universidad Javeriana Cali, Colombia. carlostororeumatologo@gmail.com. ORCID: 0000-0002-6084-7049
7. Catherine Hill, The University of Adelaide, Adelaide, South Australia, Australia 2. Department of Rheumatology, The Queen Elizabeth Hospital, Woodville South, South Australia, Australia. catherine.hill@sa.gov.au
8. Christina Duftner Department of Internal Medicine, Clinical Division of Internal Medicine II, Medical University of Innsbruck, Innsbruck, Tirol, Austria, christina.duftner@gmx.at, christina.duftner@i-med.ac.at
9. Deshiré Alpizar-Rodríguez- Division of Rheumatology, University Hospital of Geneva, 26 Ave Beau-Sejour, CH 1211 Geneva, Switzerland.
10. Dr Ran Nakashima (Sasai), Department of Rheumatology and Clinical Immunology, Graduate School of Medicine, Kyoto University, 54 Shogoin-Kawahara-cho, Sakyo-ku, Kyoto 606-8507
11. Dzifa Dey, Department of Medicine and Therapeutics, University of Ghana School of Medicine and Dentistry, College of Health Sciences, Korle-Bu, Accra, Ghana. drdzifadey@gmail.com. ORCID: 0000-0002-3339-5112.
12. Elena Nikiphorou, Centre for Rheumatic Diseases, King's College London, London, UK. 2. Rheumatology Department, King's College Hospital, London, UK. ORCID: 0000-0001-6847-3726. enikiphorou@gmail.com
13. Ghita Harifi, Rheumatology Unit, Department of Medicine, Dubai Hospital, Dubai, United Arab Emirates
14. Grace Wright, Association of Women in Rheumatology, 345 E 37th Street, Suite 303C, New York, NY 10016
https://orcid.org/0000-0002-9629-9662

15. Humeira Badsha doctorbadsha@gmail.com, info
16. Ihasane Hmmamouchi, Health Sciences Research Centre (CReSS), Morocco. 2.Faculty of Medicine, International University of Rabat (UIR), Morocco. ihsane.hmamouchi@gmail.com. ORCID: 0000-0003-4402-5034.
17. Joan Von Feldt, University of Pennsylvania, Philadelphia, PA, USA.
18. Júlia Boechat Farani, Pontifícia Universidade Católica do Rio Grande do Sul, Porto Alegre (RS), Brazil
19. Laura Andreoli, Rheumatology and Clinical Immunology Unit, ASST Spedali, Civili and University of Brescia, 25123, Brescia, Italy.
20. Laura Coates, Nuffield Department of Orthopaedics, Rheumatology and Musculoskeletal Sciences, University of Oxford, Oxford, UK
21. Laure Gossec, Sorbonne Université, INSERM, Institut Pierre Louis d'Epidémiologie et de Santé Publique, and Pitié Salpêtrière Hospital, AP-HP, Rheumatology Department, Paris, France. laure.gossec@aphp.fr
22. Lisa Traboco, St Luke's Medical Center-Global City, Philippines. 2. University of the Philippines, Manila, Philippines.
23. Mariana Peixoto Guimarães Ubirajara e Silva de Souza, Post Graduate Program in Sciences Applied to Adult Health Care, Federal University of Minas Gerais
24. Neelam Hassan, MRC Clinical Research Fellow - University of Bristol Honorary Rheumatology Registrar- North Bristol NHS Trust
25. Nelly Ziadé, Rheumatology Department, Hôtel-Dieu de France Hospital, Saint Joseph's University, Beirut, Lebanon
26. Pavel Ovseiko, Radcliffe Department of Medicine, University of Oxford, Oxford, UK.
27. Penélope Esther Palomino, Department of Rheumatology, Hospital de Clinicas de Porto Alegre, Rua Ramiro Barcelos 2350, sexto andar, Porto Alegre, 90035-903, Brazil.
28. Syed Atiqul Haq, Rheumatology, Green Life Center for Rheumatic Care and Research, Dhaka, BGD
29. Vikas Agarwal, Department of Clinical Immunology and Rheumatology, Sanjay Gandhi Postgraduate Institute of Medical Sciences, Lucknow, India. Orcid ID: 0000-0002-4508-1233.
30. Wilson Bautista-Molano, Clinical Immunology Group, Universidad Militar Nueva Granada, Bogotá, Colombia. ORCID: 0000-0003-0684-9542.
31. Yoshiya Tanaka, The First Department of Internal Medicine, School of Medicine, University of Occupational and Environmental Health, 1-1 Iseigaoka, Kitakyushu, 807-8555, Japan.
