# Supplementary material for: Advances and barriers in gender equity within Brazilian rheumatology: a two-year follow-up study
Source: Adv Rheumatol. 2026 Jul 17;66:58. doi: 10.1186/s42358-026-00566-8 (PMC13663116; doi:10.1186/s42358-026-00566-8)
Supplement: Supplementary file 2 [file 42358_2026_566_MOESM2_ESM.pdf]

Appendix 2- Reuma Equity Questionnaire

GENDER EQUITY SURVEY

Research directed at Brazilian Rheumatologists

1. I consent voluntarily to the use of the information I provide in the understanding that it will be aggregated for purposes of analysis, publications, and presentations so that my responses will not be identified or identifiable. (Mark only one option)

   O - Yes
   O - No

2. What is your gender? (Mark only one option)

   O - Female
   O - Male
   O - Other

3. How old are you (in years)? (Mark only one option)

   O – 22 to 26
   O – 27 to 31
   O – 32 to 36
   O – 37 to 41
   O – 42 to 46
   O – 47 to 51
   O – 52 to 56

O – 57 to 61
O – over 62

4. What is your self-declared race? (Mark only one option)

O – White
O – Black
O – Brown
O – Yellow
O – Indian
O - Other

5. Which region of Brazil do you work? (Mark only one option)

O – Midwest
O – North
O – Northeast
O – South
O – Southest

6. Year of graduation in medicine?

____

7. Year of completion of rheumatology specialization?

____

8. Do you have the Brazilian Rheumatology Society specialist title? (Mark only one option).

O – Yes
O – No

9. Which year did you get your Brazilian Rheumatology Society specialist title?

____

10. What is your academic background? (Mark only one option)

O – Graduation
O – Pos-graduation lato-sensu
O – Medical Residence

O – Master Degree
O – Doctorate
O – Postdoctoral

11. In your rheumatology work, to which sector do you dedicate most of your time? (Mark only one option)

O – Public
O – Private

12. What area do you work in? (Mark all which apply)

[ ] Private practice (consultation)
[ ] Private non-academic hospital
[ ] Public non-academic hospital
[ ] Academic service (consultation and/or hospital)

13. Do you have a leadership role at your institution? (Mark only one option)

O – Yes
O – No

14. What is your weekly workload? (Mark only one option)

O - <20 hours
O – 20 to 44 hours
O – 45 to 60 hours
O - > 60 hours

15. What is the average time of your rheumatologic consultation? (Mark only one option)

O - <15 min
O – 15 – 30 min
O – 30 – 45 min
O – 45 – 60 min

16. What is the average value of your rheumatologic consultation?(Mark only one option)

O - < R$100
O – R$100 to R$200
O – R$201 to R$400

O – R$401 to R$600
O – R$601 to R$800
O – R$801 to R$1.000
O - > R$ 1001
O – No private consultation

17. Do you believe that there is a remuneration gap between men and women in rheumatology? (Mark only one option)

O – Yes

O – No

O – I don’t know

18. In your work life (check the most appropriate option per line)

	I totally agree	I partially agree	I don't agree or disagree	I partially disagree	I totally disagree
Thanks to my resourcefulness, I know how to handle unforeseen situations in my job	O	O	O	O	O
If I am in trouble at my work, I can usually think of something to do	O	O	O	O	O
I can remain calm when facing difficulties in my job because I can rely on my abilities	O	O	O	O	O
When I am confronted with a problem in my job, I can usually find several solutions	O	O	O	O	O
No matter what comes my way in my job, I’m usually able to handle it	O	O	O	O	O
My past experiences in my job have prepared me well for my occupational future	O	O	O	O	O
I meet the goals that I set for myself in my job.	O	O	O	O	O
I feel prepared to meet most of the demands in my job	O	O	O	O	O

19 - Have you participated in any official **national** event of the Brazilian Society of Rheumatology? (Mark all which apply).

[ ] Speaker
[ ] Moderator or Table President
[ ] Organizing

[ ] Attendee
[ ] I have never participated

20 Have you participated in any official **regional** event of the Brazilian Society of Rheumatology? (Mark all which apply)

[ ] Speaker
[ ] Moderator or Table President
[ ] Organizing
[ ] Attendee
[ ] I have never participated

21 - What is your marital status? (Mark only one option)

O – Single
O – Married
O – Divorced
O – Widow (er)
O – Stable union
O – Other

22 If you have a partner, what is his/her work regime? (Mark only one option)

O – Full-time work
O – Part-time work
O – No paid work
O – Does not apply

23 Are you legally responsible for someone under the age of 18? (Mark only one option)

O – Yes
O – No

24 Do you have children? (Mark only one option)

O – Yes
O – No

25 Did you have maternity or paternity leave? (Mark only one option)

O - Yes, less than 4 months
O - Yes, more than 4 months

O - I am self-employed and decided to return before 4 months
O - I am self-employed and decided to return after 4 months
O - Does not apply

26 Have you or do you breastfeed your children? (Mark only one option)

O - Yes, and I had **no** change in the workload during this period
O - Yes, and I had a change in the workload during this period
O – No
O – Does not apply

27 In your work-family interface (check the most appropriate option per line)

	I totally agree	I partially agree	I don't agree or disagree	I partially disagree	I totally disagree
My family demands changes / changed the course of my career	O	O	O	O	O
I refuse / refused a career progression for family reasons	O	O	O	O	O
The demands of my work interfere with my home and family life	O	O	O	O	O
The amount of time my job takes up makes it difficult to fulfil family responsibilities	O	O	O	O	O
Things I want to do at home do not get done because of the demands my job puts on me	O	O	O	O	O
My job produces strain that makes it difficult to fulfil family duties	O	O	O	O	O
Due to work-related duties, I have / had to make changes to my plans for family activities	O	O	O	O	O

28 In your family nucleus (check the most appropriate option per line)

	Totally by men	Predominantly by men	Egalitarian between men and women	Predominantly by women	Totally by women
The household chores are performed by	O	O	O	O	O
The family care is performed by	O	O	O	O	O

The family costs and expenses are performed by	O	O	O	O	O

29 As a rheumatologist have you ever suffered some kind of gender discrimination in the following situations? (Mark all which apply)

[ ] Job interview
[ ] Academic environment
[ ] Promotion opportunity
[ ] Leadership position opportunity
[ ] I have never suffered gender discrimination
[ ] Other situation

30 Have you ever experienced one of the situations below, that you consider to be related to your gender? (Mark all which apply)

[ ] Not getting a job or promotion
[ ] Not been admitted to any workplace or academic institution
[ ] Not receiving a research grant
[ ] Not gaining a leadership position
[ ] Receiving lower remuneration to perform the same work
[ ] I have never experienced any of the above situations
[ ] Other situation

31 Have you ever experienced one of these sexual harassment forms:

	Yes	No	I am not sure
Verbal (degrading or sexualized speech, sexual innuendo, obscene language)	O	O	O
Nonverbal (whistling, staring, obscene gestures, letters, email, text message, jokes, pictures)	O	O	O
Physical (groping, attempted kissing, sexual attack, advantages for sexual favors)	O	O	O

32 - Check the most appropriate option (Mark one option per line)

	I totally disagree	I partially disagree	I don't disagree or agree	I partially agree	I totally agree
I believe there is gender inequity in my workplace	O	O	O	O	O

It is appropriate that women receive less than men to perform the same tasks since women may need to interrupt their careers at times	O	O	O	O	O
There are medical specialties where women are better paid than men	O	O	O	O	O
I believe there is gender inequity in institutions and that this affects decisions regarding job opportunities and receiving funding for research	O	O	O	O	O
I believe that there is a smaller number of women in leadership positions and that is due to gender inequity	O	O	O	O	O
It is hard to be successful in my career without sacrificing the family	O	O	O	O	O
I am satisfied with the direction of my career	O	O	O	O	O
I think it is important and necessary to create strategies for gender equity in rheumatology	O	O	O	O	O

33 Do you think these measures could have a positive impact on gender equity in rheumatology?

	I totally disagree	I partially disagree	I don't disagree or agree	I partially agree	I totally agree
Increase visibility of female role models	O	O	O	O	O
Leadership skills training for women	O	O	O	O	O
Training for career planning aiming at family and profession balance	O	O	O	O	O
Skills training in communication and public presentation	O	O	O	O	O
Skills training in high-impact scientific writing	O	O	O	O	O
Implementation of result-based compensation model including patient satisfaction	O	O	O	O	O
Promotion and salary negotiation training	O	O	O	O	O
Involving men in advancing gender equity	O	O	O	O	O
Promotion of civil society sensibilization on gender equity subject	O	O	O	O	O

Family and child-friendly congress and other events	O	O	O	O	O
Promoting gender-sensitive clinical practice, research, and training	O	O	O	O	O
Establishing a Brazilian Society of Rheumatology network for women	O	O	O	O	O
Social media campaign promoting female leadership in scientific population	O	O	O	O	O
Social media campaign promoting gender equity in civil population	O	O	O	O	O
Flexibility of working hours (WITHOUT workload reduction) to care to family needs	O	O	O	O	O
Flexibility of working hours (WITH workload reduction) to care to family needs	O	O	O	O	O
Gender-balanced Brazilian Society of Rheumatology committees, working groups, task forces, editorial boards and peer-review in the “Advances in Rheumatology” (Brazilian journal)	O	O	O	O	O
