# Supplementary material for: Advances and barriers in gender equity within Brazilian rheumatology: a two-year follow-up study
Source: Adv Rheumatol. 2026 Jul 17;66:58. doi: 10.1186/s42358-026-00566-8 (PMC13663116; doi:10.1186/s42358-026-00566-8)
Supplement: Supplementary file 3 [file 42358_2026_566_MOESM3_ESM.pdf]

Appendix 3 - Coalition for health and gender equity (CHANGE) questionnaire

Original_text	**Translated_text**
Dear participant,<br><br>The survey will help us determine the extent to which rheumatology healthcare professionals need support in career advancement, particularly as it relates to global equity. We encourage rheumatology healthcare professionals currently in practice or training to complete this survey. The survey takes only 5 minutes to complete, and all responses will be completely anonymous. By pressing the next button, you agree and give permission for us to use your anonymous responses for medical education and research purposes.<br><br>For any queries, please contact: globalgeargroup@gmail.com	Caro participante,<br><br>A pesquisa nos ajudará a determinar até que ponto os profissionais de saúde em reumatologia precisam de apoio na progressão na carreira, particularmente no que diz respeito à equidade global. Encorajamos os profissionais de saúde de reumatologia a completarem este questionário. O questionário demora apenas 5 minutos e todas as respostas serão completamente anônimas. Ao pressionar o botão seguinte, você concorda e nos dá permissão para usar suas respostas anônimas para fins de educação médica e pesquisa.<br><br>Obrigado!<br><br>Para quaisquer dúvidas, entre em contato com: globalgeargroup@gmail.com
1. Which Brazilian's region do you work? (check only one option) –<br>O – North<br>O – Northeast<br>O – Midwest<br>O – Southeast<br>O -- South	1. Qual região do Brasil você trabalha? (marque apenas uma opção) –<br>O – Norte<br>O – Nodeste<br>O – Centro- Oeste<br>O – Sudeste<br>O – Sul
As a rheumatologist/rheumatology healthcare professional, have you ever suffered some kind of gender discrimination (i.e. unequal or disadvantageous treatment) OR experienced one of the situations below, that you consider to be related to your gender?	Enquanto reumatologista, alguma vez sofreu algum tipo de discriminação de género (ou seja, tratamento desigual ou desvantajoso) OU passou por alguma das situações abaixo, que considera estar relacionada com o seu gênero?
Not getting a promotion	Não receber uma promoção
Not hired by a medical organization	Não ser contratado por uma organização médica
Not hired by an academic institution	Não ser contratado por uma instituição acadêmica
Not receiving a research grant	Não receber uma bolsa de pesquisa
Not being groomed for a leadership position	Não estar preparado para um cargo de liderança

Not getting a leadership position for which I applied	Não conseguir um cargo de liderança para o qual me candidatei
Receiving lower remuneration to perform the same work	Receber remuneração inferior para realizar o mesmo trabalho
Being asked to take care of tasks that are below my level of expertise (usually time-consuming and non-intellectual tasks)	Ser solicitado a cuidar de tarefas que estão abaixo do meu nível de especialização (geralmente tarefas demoradas e não intelectuais)
Co-worker's behavior creating a hostile or intimidating environment without a sexual connotation	Comportamento do colega de trabalho criando um ambiente hostil ou intimidante sem conotação sexual
Never	Nunca
Rarely	Raramente
Sometimes	Às vezes
Often	Muitas vezes
Always	Sempre
Other (please specify)	Outros (especificar)
This question requires an answer.	Esta pergunta requer uma resposta.
Please tell us if you have personally experienced gender discrimination (i.e., unequal or disadvantageous treatment) from colleagues or others during your professional career.	Diga-nos se sofreu pessoalmente discriminação de gênero (ou seja, tratamento desigual ou desvantajoso) por parte de colegas ou outras pessoas durante a sua carreira profissional.
Yes, from older colleagues	Sim, de colegas mais velhos
Yes, from younger colleagues	Sim, de colegas mais jovens
Yes, from nurses	Sim, de enfermeiros (as)
Yes, from patients	Sim, de doentes
Yes, from administrators	Sim, de administradores (as)
Yes, from secretaries	Sim, de secretários (as)
Yes, from students	Sim, de estudantes
Yes, from others in the working environment	Sim, de outras pessoas no ambiente de trabalho
Never	Nunca

Rarely	Raramente
Sometimes	Às vezes
Often	Muitas vezes
Always	Sempre
This question requires an answer.	Esta pergunta requer uma resposta.
Please tell us if you have personally experienced sexual harassment (i.e., unwanted behavior of a sexual nature) from colleagues or others during your professional career.	Por favor, diga-nos se você sofreu pessoalmente assédio sexual (ou seja, comportamento indesejado de natureza sexual) de colegas ou outras pessoas durante sua carreira profissional.
Yes, from older colleagues	Sim, de colegas mais velhos (as)
Yes, from younger colleagues	Sim, de colegas mais jovens
Yes, from nurses	Sim, de enfermeiros (as)
Yes, from patients	Sim, de doentes
Yes, from administrators	Sim, de administradores (as)
Yes, from secretaries	Sim, de secretários (as)
yes, from students	Sim, de estudantes
Yes, from others in the working environment	Sim, de outras pessoas no ambiente de trabalho
Never	Nunca
Rarely	Raramente
Sometimes	Às vezes
Often	Muitas vezes
Always	Sempre
This question requires an answer.	Esta pergunta requer uma resposta.
Have you ever personally experienced sexual harassment during your career?	Você já sofreu assédio sexual pessoalmente durante sua carreira?
I have never experienced it	Eu nunca experimentei isso
Ongoing	Em curso

Experienced in the past	Experiente no passado
This question requires an answer.	Esta pergunta requer uma resposta.
Have you ever felt bullied at work?	Você já se sentiu intimidado(a) no trabalho?
Never	Nunca
Rarely	Raramente
Sometimes	Às vezes
Often	Muitas vezes
Always	Sempre
This question requires an answer.	Esta pergunta requer uma resposta.
Please tell us about the nature of bullying.	Por favor, fale-nos sobre a natureza da intimidação.
I have never experienced it	Eu nunca experimentei isso
Ongoing	Em curso
Experienced in the past	Experiente no passado
This question requires an answer.	Esta pergunta requer uma resposta.
Please mark all sentences that apply to yourself.	Por favor, marque todas as frases que se aplicam a si mesmo(a).
Thanks to my resourcefulness (personality), I know how to handle unforeseen situations in my job.	Graças à minha desenvoltura (personalidade), sei lidar com imprevistos no meu trabalho.
If I am in trouble at my work, I can usually think of something to do.	Se estou com problemas no meu trabalho, geralmente consigo pensar em algo para fazer.
I can remain calm when facing difficulties in my job because I can rely on my abilities.	Posso manter a calma quando enfrento dificuldades no meu trabalho, porque posso confiar nas minhas capacidades.
When I am confronted with a problem in my job, I can usually find several solutions.	Quando sou confrontado com um problema no meu trabalho, normalmente consigo encontrar várias soluções.
No matter what comes my way in my job, I'm usually able to handle it.	Não importa o que aconteça no meu trabalho, geralmente sou capaz de lidar com isso.
My past experiences in my job have prepared me well for my occupational future.	As minhas experiências passadas no meu trabalho prepararam-me bem para o meu futuro profissional.

I meet the goals that I set for myself in my job.	Cumpro os objetivos que estabeleci para mim no meu trabalho.
I feel prepared to meet most of the demands in my job.	Sinto-me preparado para satisfazer a maioria das exigências do meu trabalho.
This question requires an answer.	Esta pergunta requer uma resposta.
In your family nucleus, how are the household chores and family care responsibilities divided among men and women?	No seu núcleo familiar, como são divididas as tarefas domésticas e as responsabilidades familiares entre homens e mulheres?
Equally shared between men and women	Igualmente partilhadas entre homens e mulheres
Predominantly performed by men	Predominantemente realizadas por homens
Predominantly performed by women	Predominantemente realizadas por mulheres
Does not apply	Não se aplica
This question requires an answer.	Esta pergunta requer uma resposta.
Please rate your level of agreement with the following statements as it relates to your reality.	Por favor, classifique o seu nível de concordância com as seguintes afirmações em relação à sua realidade.
My work demands have a negative impact on family activities and planning (like social events or vacations)	As minhas exigências de trabalho têm um impacto negativo nas atividades familiares e no planejamento (como eventos sociais ou férias)
My work demands have a negative impact on my household duties (like house chores)	As minhas exigências de trabalho têm um impacto negativo nas minhas tarefas domésticas
My family demands have a negative impact on my work responsibilities and duties	As minhas exigências familiares têm um impacto negativo nas minhas responsabilidades e deveres profissionais
Strongly disagree	Discordo totalmente
Disagree	Discordo
Neutral	Neutro
Agree	Concordo
Strongly agree	Concordo totalmente
This question requires an answer.	Esta pergunta requer uma resposta.

Do you believe that having children impacted the course of your career?	Acredita que ter filhos impactou o rumo da sua carreira?
Yes, positive impact	Sim, impacto positivo
Yes, negative impact	Sim, impacto negativo
Yes, both positive and negative impact	Sim, impacto positivo e negativo
No impact	Sem impacto
Does not apply	Não se aplica
This question requires an answer.	Esta pergunta requer uma resposta.
Do you believe that your work may have influenced your family planning, for example, having children or having multiple children?	Acredita que o seu trabalho pode ter influenciado o seu planeamento familiar, por exemplo, ter filhos ou ter vários filhos?
Yes	Sim
No	Não
I am not sure	Não tenho a certeza
Does not apply	Não se aplica
This question requires an answer.	Esta pergunta requer uma resposta.
Have you refused any career advancement opportunities due to family reasons?	Recusou qualquer oportunidade de progressão na carreira por razões familiares?
Yes	Sim
No	Não
I am not sure	Não tenho a certeza
Does not apply	Não se aplica
This question requires an answer.	Esta pergunta requer uma resposta.
Please select the statements that apply to your current career situation.	Por favor, selecione as declarações que se aplicam à sua situação profissional atual.
I am actively seeking promotion at my workplace.	Procuro ativamente promoção no meu local de trabalho.
I desire to be promoted to a leadership role or to a greater leadership role.	Desejo ser promovido a um papel de liderança ou a um papel de liderança maior.

I feel/felt ready for a promotion to a leadership role or to a greater leadership role.	Sinto-me pronto para uma promoção a um papel de liderança ou a um papel de liderança maior que o atual.
I intend to seek a sabbatical year (research leave) from my current position.	Pretendo buscar um ano sabático (licença de pesquisa) do meu cargo atual.
I intend to voluntarily step down from my current leadership role or career roles in the next 1-2 years.	Pretendo demitir-me voluntariamente do meu atual cargo de liderança ou carreira nos próximos 1-2 anos.
I intend to leave my current place of work for a position in rheumatology elsewhere in the next 1-2 years.	Pretendo deixar o meu local de trabalho atual para um cargo em reumatologia noutro local nos próximos 1-2 anos.
I intend to leave rheumatology in the next 1–2 years.	Pretendo deixar a reumatologia nos próximos 1 a 2 anos.
None of these apply	Nenhuma destas situações se aplica
This question requires an answer.	Esta pergunta requer uma resposta.
Please indicate which work-based interventions would be helpful for your career advancement. (Mark all which apply)	Indique quais as intervenções no trabalho seriam úteis para a sua progressão na carreira. (Marque todos os que se aplicam)
Peer-to-peer mentorship programmes	Programas de mentoria entre pares
Senior sponsorship programmes	Programas de patrocínio sênior
Personality insights, e.g. Insights Discovery®	Teste vocacional com programas que auxiliam as pessoas a superarem os desafios críticos para o sucesso individual como, por exemplo, Insights Discovery®
Raising awareness of gender equity issues in rheumatology	Sensibilização para as questões de igualdade de gênero na reumatologia
Involving men in advancing gender equity	Envolver os homens na promoção da equidade de gênero
Increasing visibility of female role models	Aumentar a visibilidade dos modelos femininos
Implementation of result-based compensation model including patient satisfaction	Implementação de modelo de remuneração baseado em resultados, incluindo satisfação do paciente
Promotion of civil society sensibilization on gender equity subject	Promoção da sensibilização da sociedade civil sobre o tema da equidade de gênero

Flexibility of working hours (WITHOUT workload reduction) to care to family needs	Flexibilidade de horários de trabalho (SEM redução da carga de trabalho) para atender às necessidades da família
Flexibility of working hours (WITH workload reduction) to care to family needs	Flexibilidade de horários de trabalho (COM redução da carga horária) para atender às necessidades da família
Please enter a comment.	Por favor, insira um comentário.
Other (please specify)	Outros (especificar)
This question requires an answer.	Esta pergunta requer uma resposta.
Please indicate which interventions related to skills training would be helpful for your career advancement. (Mark all which apply)	Por favor, indique quais intervenções relacionadas a treinamento de habilidades seriam úteis para o seu avanço na carreira. (Marque todos os que se aplicam)
Leadership skills training	Formação em competências de liderança
Speaking, scientific writing, presentation, communication skills training	Expressão oral, escrita científica, apresentação, formação em competências de comunicação
Effective training on career planning/pathways	Formação eficaz em planejamento/estratégias de carreira
Promotion and salary negotiation training	Formação em promoção e negociação salarial
Support on grant writing applications	Apoio na escrita de formulários de solicitação de verba
High-impact scientific writing masterclasses	Aulas de escrita científica de alto impacto
Effective work-life balance management training	Formação eficaz em gestão do equilíbrio entre a vida profissional e a vida familiar
Unconscious bias and/or implicit bias training (expose people to their implicit biases, provide tools to adjust automatic patterns of thinking, and ultimately eliminate discriminatory behaviors)	Treinamento de viés inconsciente e/ou preconceito implícito (expor as pessoas a seus vieses implícitos, fornecer ferramentas para ajustar padrões automáticos de pensamento e, finalmente, eliminar comportamentos discriminatórios)
Promoting gender-sensitive clinical practice, research, and training	Promover de forma sensível ao gênero, a prática clínica, a investigação e a formação.
This question requires an answer.	Esta pergunta requer uma resposta.
Please indicate which conference-related interventions would be	Indique quais as intervenções relacionadas às conferências/ eventos seriam úteis para a sua

helpful for your career advancement. (Mark all which apply)	progressão na carreira. (Marque todos os que se aplicam)
No male-only panel pledge at rheumatology conferences and events	Evitar painéis exclusivamente masculinos em conferências e eventos de reumatologia
Family- and child-friendly rheumatology conferences and events	Conferências e eventos de reumatologia com permissão para famílias e crianças
This question requires an answer.	Esta pergunta requer uma resposta.
Please indicate which interventions related to career advancement would be helpful for your career advancement. (Mark all which apply)	Indique quais as intervenções seriam úteis para a sua progressão na carreira. (Marque todos os que se aplicam)
Establishing a Society/Network of Gender equity in Rheumatology (GEAR)	Estabelecimento de uma Sociedade/Rede de trabalho para Igualdade de Gênero em Reumatologia (GEAR)
#SheLeadsRheumatology or similar social media campaign	#SheLeadsRheumatology (ela lidera a reumatologia) ou campanha semelhante nas redes sociais
Gender-balanced committees, working groups, and task-forces	Comitês, grupos de trabalho e forças tarefas equilibrados em termos de gênero
Policies on gender-balanced organizational funding	Políticas de financiamento organizacional equilibrado em termos de gênero
Gender balanced editorial boards and peer-review in rheumatology journals	Conselhos editoriais e revisão por pares em revistas de reumatologia equilibrados em termos de gênero
Engaging national rheumatology groups to develop country-specific support	Envolver grupos nacionais de reumatologia para desenvolver apoio específico a cada país
Social media campaign promoting female leadership in the scientific population	Campanha nas redes sociais para promover a liderança feminina na população científica
Social media campaign promoting gender equity in the civil population	Campanha nas redes sociais para promover a igualdade de gênero na população civil
This question requires an answer.	Esta pergunta requer uma resposta.
Please tell us about your career	Fale-nos sobre a sua carreira
Which of the following best describes your current job role? (Mark all which apply)	Qual das seguintes opções melhor descreve a sua função atual? (Marque todos os que se aplicam)
Student	Estudante

Practicing physician rheumatologist	Médico reumatologista praticante
Non-clinical academic/researcher	Acadêmico/investigador não clínico
Clinical academic/researcher	Acadêmico/investigador clínico
Administrator	Administrador
Rheumatology Healthcare Professional	Profissional de Saúde em Reumatologia
Please enter a comment.	Por favor, insira um comentário.
Other (please specify)	Outros (especificar)
This question requires an answer.	Esta pergunta requer uma resposta.
Are you employed full-time or less than full-time?	Tem emprego de tempo integral ou parcial?
Full time	Tempo integral
Less than full time	Tempo parcial
This question requires an answer.	Esta pergunta requer uma resposta.
What are your typical working hours in a week?	Qual a sua carga horária de trabalho em uma semana (em horas)?
Please enter a whole number between {0} and {1}.	Introduza um número inteiro entre {0} e {90}.
0	0
90	90
This question requires an answer.	Esta pergunta requer uma resposta.
Do you have a leadership role (e.g., group leader, head of the department, chief of service, other organizational leadership positions, etc.)?	Possui um papel de liderança (por exemplo, líder de grupo, chefe de departamento, chefe de serviço, outros cargos de liderança organizacional)?
Yes, in an academic setting	Sim, em ambiente acadêmico
Yes, in a society	Sim, numa sociedade
Yes, in a hospital	Sim, num hospital
No	Não
This question requires an answer.	Esta pergunta requer uma resposta.

Do you currently have an academic role? If so, what is it?	Tem formação acadêmica adicional? Em caso afirmativo, qual é?
No	Não
Yes - PhD student	Sim - Estudante de mestrado ou doutorado
Yes - Postdoc	Sim - Pós-doc
Yes - Assistant Professor/Associate professor or similar	Sim - Professor Assistente/Professor Associado ou similar
Yes - Professor	Sim - Professor Titular
Please enter a comment.	Por favor, insira um comentário.
Other (please specify)	Outros (especificar)
This question requires an answer.	Esta pergunta requer uma resposta.
In your rheumatology work, to which sector do you dedicate most of your time?	No seu trabalho de reumatologia, a qual setor dedica a maior parte do seu tempo?
Public	Público
Private	Privado
I devote equal time to both sectors	Dedico tempo semelhante a ambos os setores
This question requires an answer.	Esta pergunta requer uma resposta.
How many years have you been in practice (since graduation as a medical student)?	Há quantos anos você está na prática médica (desde que se formou como estudante de medicina)?
Please enter a whole number between {0} and {1}.	Introduza um número inteiro entre {0} e {80}.
0	0
80	80
This question requires an answer.	Esta pergunta requer uma resposta.
What is the average time you spend during a rheumatology patient consultation for new cases (in minutes)?	Qual é o tempo médio que você gasta durante uma primeira consulta reumatológica (novos casos) (em minutos)?
Please enter a whole number between {0} and {1}.	Introduza um número inteiro entre {0} e {60}.
60	60

0	0
This question requires an answer.	Esta pergunta requer uma resposta.
What is the average time you spend during a rheumatology patient consultation for follow-up cases (in minutes)?	Qual é o tempo médio que você gasta durante uma consulta reumatológica de seguimento (casos de acompanhamento) (em minutos)?
Please enter a whole number between {0} and {1}.	Introduza um número inteiro entre {0} e {1}.
60	60
0	0
This question requires an answer.	Esta pergunta requer uma resposta.
What is the average value of your rheumatologic consultation (in USD)?<br>Please select 0 if you work in the government/ public sector.	Qual é o valor médio da sua consulta reumatológica (em USD)?<br>Por favor, selecione 0 se você trabalha no governo / setor público.
Please enter a whole number between {0} and {1}.	Introduza um número inteiro entre {0} e {1000}.
1000	1000
0	0
This question requires an answer.	Esta pergunta requer uma resposta.
Have you participated in an official event of a Rheumatology society at the local/regional, national, or international level?	Você participou de um evento oficial de uma sociedade de Reumatologia a nível local/regional, nacional ou internacional?
Yes	Sim
No	Não
This question requires an answer.	Esta pergunta requer uma resposta.
Please tell us about your career	Fale-nos sobre a sua carreira
In what role have you participated in an official event of a Rheumatology society at the local/regional, national or international level? (Mark all which apply)	Em qual função participou de um evento oficial de uma sociedade de Reumatologia a nível local/regional, nacional ou internacional? (Marque todos os que se aplicam)
Invited Speaker	Palestrante Convidado

Moderator or Table President	Moderador ou Presidente da Mesa
Organizing President of the event	Presidente organizador do evento
Presenter of an abstract	Apresentação de poster
Attendee	Participante / ouvinte
Local/Regional level	Nível local/regional
National level	Nível nacional
International level	Nível internacional
This question requires an answer.	Esta pergunta requer uma resposta.
Please tell us about yourself	Por favor, fale-nos sobre você
In what country do you work?	Em que país trabalha?
Afghanistan	Afeganistão
Albania	Albânia
Algeria	Argélia
Andorra	Andorra
Angola	Angola
Antigua and Barbuda	Antígua e Barbuda
Argentina	Argentina
Armenia	Arménia
Australia	Austrália
Austria	Áustria
Azerbaijan	Azerbaijão
Bahamas	Baamas
Bahrain	Bahrein
Bangladesh	Bangladeche
Barbados	Barbados
Belarus	Bielorrússia

Belgium	Bélgica
Belize	Belize
Benin	Benim
Bhutan	Butão
Bolivia (Plurinational State of)	Bolívia (Estado Plurinacional de)
Bosnia and Herzegovina	Bósnia e Herzegovina
Botswana	Botsuana
Brazil	Brasil
Brunei Darussalam	Brunei Darussalam
Bulgaria	Bulgária
Burkina Faso	Burquina Faso
Burundi	Burundi
Cabo Verde	Cabo Verde
Cambodia	Camboja
Cameroon	Camarões
Canada	Canadá
Central African Republic	República Centro-Africana
Chad	Chade
Chile	Chile
China	China
Colombia	Colômbia
Comoros	Comores
Congo	Congo
Costa Rica	Costa Rica
Côte D'Ivoire	Costa do Marfim
Croatia	Croácia

Cuba	Cuba
Cyprus	Chipre
Czech Republic	República Checa
Democratic People's Republic of Korea	República Popular Democrática da Coreia
Democratic Republic of the Congo	República Democrática do Congo
Denmark	Dinamarca
Djibouti	Djibuti
Dominica	Domínica
Dominican Republic	República Dominicana
Ecuador	Equador
Egypt	Egito
El Salvador	El Salvador
Equatorial Guinea	Guiné Equatorial
Eritrea	Eritreia
Estonia	Estónia
Ethiopia	Etiópia
Fiji	Fiji
Finland	Finlândia
France	França
Gabon	Gabão
Gambia	Gâmbia
Georgia	Geórgia
Germany	Alemanha
Ghana	Gana
Greece	Grécia
Grenada	Granada

Guatemala	Guatemala
Guinea	Guiné
Guinea Bissau	Guiné-Bissau
Guyana	Guiana
Haiti	Haiti
Holy See	Santa Sé
Honduras	Honduras
Hong Kong SAR	Região Administrativa Especial de Hong Kong
Hungary	Hungria
Iceland	Islândia
India	Índia
Indonesia	Indonésia
Iran (Islamic Republic of)	Irão (República Islâmica do)
Iraq	Iraque
Ireland	Irlanda
Israel	Israel
Italy	Itália
Jamaica	Jamaica
Japan	Japão
Jordan	Jordânia
Kazakhstan	Cazaquistão
Kenya	Quénia
Kiribati	Quiribáti
Kuwait	Kuwait
Kyrgyzstan	Quirguizistão
Lao People’s Democratic Republic	República Democrática Popular do Laos

Latvia	Letónia
Lebanon	Líbano
Lesotho	Lesoto
Liberia	Libéria
Libya	Líbia
Liechtenstein	Liechtenstein
Lithuania	Lituânia
Luxembourg	Luxemburgo
Madagascar	Madagáscar
Malawi	Maláui
Malaysia	Malásia
Maldives	Maldivas
Mali	Mali
Malta	Malta
Marshall Islands	Ilhas Marshall
Mauritania	Mauritânia
Mauritius	Maurícia
Mexico	México
Micronesia (Federated States of)	Micronésia (Estados Federados da)
Monaco	Mónaco
Mongolia	Mongólia
Montenegro	Montenegro
Morocco	Marrocos
Mozambique	Moçambique
Myanmar	Mianmar
Namibia	Namíbia

Nauru	Nauru
Nepal	Nepal
Netherlands	Países Baixos
New Zealand	Nova Zelândia
Nicaragua	Nicarágua
Niger	Níger
Nigeria	Nigéria
Norway	Noruega
Oman	Omã
Pakistan	Paquistão
Palau	Palau
Panama	Panamá
Papua New Guinea	Papuásia-Nova Guiné
Paraguay	Paraguai
Peru	Peru
Philippines	Filipinas
Poland	Polónia
Portugal	Portugal
Qatar	Catar
Republic of Korea	República da Coreia
Republic of Moldova	República da Moldávia
Romania	Roménia
Russian Federation	Federação Russa
Rwanda	Ruanda
Saint Kitts and Nevis	São Cristóvão e Neves
Saint Lucia	Santa Lúcia

Saint Vincent and the Grenadines	São Vicente e Granadinas
Samoa	Ilhas Samoa
San Marino	São Marinho
Sao Tome and Principe	São Tomé e Príncipe
Saudi Arabia	Arábia Saudita
Senegal	Senegal
Serbia	Sérvia e Montenegro
Seychelles	Seicheles
Sierra Leone	Serra Leoa
Singapore	Singapura
Slovakia	Eslováquia
Slovenia	Eslovénia
Solomon Islands	Ilhas Salomão
Somalia	Somália
South Africa	África do Sul
South Sudan	Sudão do Sul
Spain	Espanha
Sri Lanka	Sri Lanca
State of Palestine	Estado da Palestina
Sudan	Sudão
Suriname	Suriname
Swaziland	Suazilândia
Sweden	Suécia
Switzerland	Suíça
Syrian Arab Republic	República Árabe Síria
Taiwan	Taiwan

Tajikistan	Tajiquistão
Thailand	Tailândia
The former Yugoslav Republic of Macedonia	Antiga República jugoslava da Macedónia
Timor-Leste	Timor-Leste
Togo	Togo
Tonga	Tonga
Trinidad and Tobago	Trindade e Tobago
Tunisia	Tunísia
Turkey	Turquia
Turkmenistan	Turquemenistão
Tuvalu	Tuvalu
Uganda	Uganda
Ukraine	Ucrânia
United Arab Emirates	Emirados Árabes Unidos
United Kingdom of Great Britain and Northern Ireland	Reino Unido da Grã-Bretanha e da Irlanda do Norte
United Republic of Tanzania	República Unida da Tanzânia
United States of America	Estados Unidos da América
Uruguay	Uruguai
Uzbekistan	Usbequistão
Vanuatu	Vanuatu
Venezuela (Bolivarian Republic of)	Venezuela (República Bolivariana da)
Vietnam	Vietname
Yemen	Iémen
Zambia	Zâmbia
Zimbabwe	Zimbabué
Please enter a comment.	Por favor, insira um comentário.

Other (please specify)	Outros (especificar)
This question requires an answer.	Esta pergunta requer uma resposta.
Please tell us about yourself	Por favor, fale-nos sobre você
What is your gender?	Qual é o seu gênero?
Female (Cisgender woman)	Feminino (mulher cisgênero)
Male (Cisgender man)	Masculino (homem cisgênero)
Transgender woman	Mulher transexual
Transgender man	Homem transgênero
Non-binary person	Pessoa não-binária
Prefer not to say	Prefiro não dizer
Please enter a comment.	Por favor, insira um comentário.
Other (please specify)	Outros (especificar)
This question requires an answer.	Esta pergunta requer uma resposta.
Please tell us about yourself	Por favor, fale-nos sobre você
What is your age?	Qual é a tua idade?
Please enter a whole number between {0} and {1}.	Introduza um número inteiro entre {20} e {100}.
20	20
100	100
This question requires an answer.	Esta pergunta requer uma resposta.
Do you identify yourself as	Identifica-se como
Heterosexual/ Straight	Heterossexual/ Hétero
Lesbian	Lésbica
Gay	Gay
Bisexual	Bissexual
Queer	Queer
Prefer not to say	Prefiro não dizer

Please enter a comment.	Por favor, insira um comentário.
Other (please specify)	Outros (especificar)
This question requires an answer.	Esta pergunta requer uma resposta.
If you have a spouse/partner, what is their employment status?	Se tem um cônjuge/companheiro (a), qual é a situação profissional dele (a)?
No, I do not have a spouse/partner	Não, não tenho cônjuge/companheiro(a)
Yes, I do have a spouse/partner and they are in full-time employment	Sim, tenho um cônjuge/companheiro(a) e ele(a) trabalha em tempo integral.
Yes, I do have a spouse/partner and they are in less than full-time employment	Sim, tenho um cônjuge/companheiro(a) e ele(a) trabalha em tempo parcial.
Yes, I do have a spouse/partner and they are unemployed/ a stay at home parent	Sim, tenho um cônjuge/companheiro(a) e ele(a) não trabalha fora ou está desempregado(a).
This question requires an answer.	Esta pergunta requer uma resposta.
Do you have regular caring responsibilities for children (under the age of 18), partner, elderly or disabled relatives? (Mark all that apply)	Você é responsável pelo cuidado/custeio de: crianças abaixo de 18 anos, companheiro, idosos ou familiares com deficiência? (Marque todas as opções aplicáveis)
Yes, for children (single parent)	Sim, por crianças (famílias monoparentais)
Yes, for children (NOT as a single parent)	Sim, por crianças (juntamente com um cônjuge)
Yes, for children (divorced/ non co-inhabitant parent)	Sim, por crianças (pais divorciados/não co-habitantes)
Yes, for partner	Sim, por parceiro(a)
Yes, for elderly	Sim, por idosos
Yes, for disabled relatives	Sim, por familiares com deficiência
No	Não
This question requires an answer.	Esta pergunta requer uma resposta.
Did you ever take maternity or paternity leave?	Alguma vez tirou licença de maternidade ou paternidade?
Yes, less than 4 months	Sim, menos de 4 meses
Yes, more than 4 months	Sim, mais de 4 meses

I am self-employed and decided to return before 4 months	Sou trabalhador(a) independente e decidi regressar antes de 4 meses
I am self-employed and decided to return after 4 months	Sou trabalhador(a) independente e decidi regressar depois de 4 meses
Does not apply	Não se aplica
This question requires an answer.	Esta pergunta requer uma resposta.
How would you best describe yourself?	Qual seria a melhor forma de se descrever (etnia)?
Asian	Asiático
Indian	Indiana
Caucasian	Caucasiana
African American	Afro-americano
African	Africana
Arab	Árabe
Hispanic	Hispânica
Mixed ethnicity	Etnia mista
Prefer not to say	Prefiro não dizer
Please enter a comment.	Por favor, insira um comentário.
Other (please specify)	Outros (especificar)
This question requires an answer.	Esta pergunta requer uma resposta.
Would you like us to contact you regarding future surveys? If yes, please provide your email address.<br><br>Your email address will be strictly kept confidential and for university records only.	Gostaria que o contactássemos para futuros inquéritos? Se sim, por favor, forneça seu endereço de e-mail.<br><br>Seu endereço de e-mail será estritamente mantido confidencial e apenas para registros universitários.
Yes	Sim
No	Não
Thank you for completing our survey!	Obrigado por responder ao nosso questionário!
