# Supplementary material for: Advances and barriers in gender equity within Brazilian rheumatology: a two-year follow-up study
Source: Adv Rheumatol. 2026 Jul 17;66:58. doi: 10.1186/s42358-026-00566-8 (PMC13663116; doi:10.1186/s42358-026-00566-8)
Supplement: Supplementary file 4 [file 42358_2026_566_MOESM4_ESM.pdf]

Appendix 4 - Proposals for interventions promoting gender equity (agreement result), by gender, across studies.

	**Reuma Equity**			**Change**		
	**Women (%)**	**Men (%)**	**p-value**	**Women (%)**	**Men (%)**	**p-value**
Stablish a task force for women in the SBR	235 (76.3)	79 (52.3)	**<0.001**	83 (37.1)	21 (27.3)	0.119
Gender-balanced composition of leadership positions in the SBR and the Brazilian scientific journal "AIR"	244 (79.2)	81 (53.6)	**<0.001**	125 (55.8)	32 (41.6)	**0.031**
SBR media campaign to promote female leadership	239 (77.6)	80 (53.0)	**<0.001**	80 (35.7)	14 (18.2)	**0.004**
A media campaign to promote gender equality in the civilian population	252 (81.8)	106 (70.2)	**0.018**	92 (41.1)	28 (36.4)	0.467
Conferences that welcome families and children	220 (71.4)	82 (54.3)	**0.001**	109 (48.7)	37 (48.1)	0.927
Leadership skills training exclusively for women	220 (71.4)	62 (41.1)	**<0.001**	120 (53.6)	40 (51.9)	0.805
High-impact scientific writing training	247 (80.2)	87 (57.6)	**<0.001**	114 (50.9)	46 (59.7)	0.18
Salary negotiation and promotion training	272 (88.3)	111 (73.5)	**<0.001**			
Career planning training focusing on work-life balance	268 (87.0)	118 (78.1)	**0.007**	108 (48.2)	25 (32.5)	**0.016**
Promoting clinical practice. research. and training with a gender perspective	224 (72.8)	73 (48.3)	**<0.001**	65 (29.0)	22 (28.6)	0.941
I believe it is important and necessary to create strategies for gender equality in rheumatology	243 (78.9)	105 (69.5)	**0.022**	60 (26.8)	13 (16.9)	0.08
Men's involvement in the gender equality task force	260 (84.4)	112 (74.2)	**0.001**	61 (27.2)	10 (13.0)	**0.011**
Increase the visibility of female models	233 (75.7)	75 (49.7)	**<0.001**	88 (39.3)	12 (15.6)	**<0.001**
Implementation of a appointment-based reimbursement model that includes patient satisfaction	243 (78.9)	91 (60.3)	**<0.001**	116 (51.8)	35 (45.5)	0.338
Greater awareness among the general population regarding the issue of gender equality	261 (84.7)	107 (70.9)	**<0.001**	67 (29.9)	13 (16.9)	**0.026**
Flexible work schedule (WITHOUT reduced working hours) for family needs	230 (74.7)	106 (70.2)	**0.045**	94 (42.0)	28 (36.4)	0.388

Flexible work schedule (WITH reduced working hours) for family needs	196 (63.6)	74 (49.0)	**<0.001**	77 (34.4)	16 (20.8)	**0.026**
Self-awareness tools for behavioral profiling, such as Insights Discovery®				33 (14.7)	11 (14.3)	0.924
Sponsorship programs by senior professionals				52 (23.2)	23 (29.9)	0.244
#SheLeadsRheumatology or similar social media campaign				67 (29.9)	8 (10.4)	**<0.001**
Gender-balanced organizational funding policies				90 (40.2)	23 (29.9)	0.107
Gender-balanced editorial and peer review committees in rheumatology journals				82 (36.6)	23 (29.9)	0.285
Engagement of national rheumatology groups to develop country-specific support				98 (43.8)	39 (50.6)	0.294
Commitment to the absence of all-male panels at rheumatology congresses and events				155 (69.2)	51 (66.2)	0.629
Effective training in career planning and pathways				125 (55.8)	42 (54.5)	0.848
Salary negotiation and promotion training				78 (34.8)	28 (36.4)	0.807
Support in preparing grant writing proposals				28 (12.5)	17 (22.1)	**0.042**
Advanced classes in high-impact scientific writing				66 (29.5)	30 (39.0)	0.123
Training on unconscious and/or implicit biases				67 (29.9)	17 (22.1)	0.186
Peer mentoring programs				88 (39.3)	27 (35.1)	0.511

Abbreviations: SBR: Brazilian Society of Rheumatology, AIR: Advances in Rheumatology
